# The Psychology of Sustainable Seafood Consumption: A Comprehensive Approach

**DOI:** 10.3390/foods6100086

**Published:** 2017-09-28

**Authors:** Isabel G. M. Richter, Christian A. Klöckner

**Affiliations:** Department of Psychology, Faculty of Social and Educational Sciences, Norwegian University of Science and Technology, 7091 Trondheim, Norway; christian.klockner@ntnu.no

**Keywords:** psychology, seafood, consumer, sustainable

## Abstract

This paper discusses conceptual confusions of sustainable seafood consumption, practical challenges, and potential anchors from where this behaviour can be fostered. The main focus lies on psychological variables. The resulting framework comprises (1) a definition of sustainable seafood consumption, (2) suggestions for corresponding behaviours, (3) the identification of facilitating and hindering factors, (4) an assemblage of these factors into a theoretical model, and (5) a short discussion of how the model adds up value to the current state of the art in marine resource conservation. Behavioural models significantly contribute to behavioural change research. The originality and value of this research are that it tackles the so far relatively neglected field of sustainable seafood consumption as important part of sustainable development and marine conservation in the future. From an interventional perspective, the developed model facilitates the identification of contact points to approach consumers and disseminate sustainable seafood consumption among modern Western consumers.

## 1. Introduction

Life on earth depends on services provided by marine ecosystems [1]. However, increasing levels of marine resource depletion, ocean acidification, pollution, noise, and overfishing cause a cycle of marine degradation which in turn affects marine ecosystems and the services humanity derives from them [2]. 

This work focuses on marine resource exploitation for seafood, identified as one of the main threats to marine ecosystems [3,4,5]. Currently, more than 90% of world’s fish stocks are fully exploited, overexploited, or collapsed [6], potentially leading to the end of commercial fishing, as it is known today, in the middle of this century [7,8]. The way seafood is used needs to undergo profound changes. Otherwise, the potential of seafood as an inherently renewable protein source, that could help to feed the growing world population might be lost [9,10,11,12]. 

Seafood production and consumption skyrocketed over the last century, rising above 20.0 kilo per capita per year on a global average and over 22.0 kilo Europe [13]. This exceeds the limits of sustainable resource use in many parts of the world, especially in regards to fisheries relying on wild catches [6,14]. Besides steadily increasing consumption, another aspect makes current seafood consumption problematic as well. Particularly in western countries, the culture of seafood consumption builds around a small number of species that are commonly consumed. Unfortunately, the consumer’s favourites all come with substantial concerns in regards to sustainable fishing or fish farming [9,15]. The reason for these developments is a complex interplay of demand, supply, technical improvements, and the striving for economic efficiency [14,16,17,18,19]. Successful changes toward more sustainability can only be asserted if interdependencies of all the drivers are considered [20,21,22]. Furthermore, a stringent and comprehensive definition of sustainable seafood is brought forward that can be applied within all relevant sectors like legislation, production, and consumption. In this work, the focus lies on consumers as significant actors in marine resource use. However, we do not treat consumers as isolated from the system. We see consumers are bottom-up drivers of system change whose actions depend on the socioeconomic circumstances they are in. The significance of consumers as change agents becomes apparent by taking a global perspective. Consumer behaviour is one of the main drivers behind global resource exploitation [23,24]. Despite growing interest in sustainability and environmental protection by Western consumers, substantial behaviour change among consumers is still rare [25,26]. 

To move away from an unstainable way of consuming seafood, we propose the following steps. The first step is to shine some light on the fuzzy concept of sustainable seafood consumption and propose actions that on the one hand correspond to the criteria of sustainable seafood and on the other hand are simple to implement for the consumer. The second and third steps are finding out why these behaviours are not performed already and how these barriers can be overcome. In environmental psychology, the second and third step are often carried out with the help of models. 

## 2. Concept of Sustainable Seafood Consumption

Sustainable consumption is a broad concept that integrates various behaviours. On a “high level approach”, sustainable consumption can be defined as stated in the Oslo Symposium on Sustainable Consumption in 1994: “the use of goods and services that respond to basic needs and bring a better quality of life, while minimizing the use of natural resources, toxic materials and emissions of waste and pollutants over the life cycle, so as not to jeopardize the needs of future generations” [27]. However, this definition does not provide information for the consumer about which behaviours are considered sustainable or unsustainable in the consumption situation. Some institutions like the Department for the Environment, Food and Rural Affairs in the UK, provide lists with behavioural suggestions for consumers to cover this gap [28]. Still these suggestions usually do not become very specific in reagrds to sustainbale seafood consumption. Sustainable seafood can be understood as “seafood fished or farmed in a manner that can maintain or increase production in the long term, without jeopardising the health or function of the web of life in our oceans” [29]. In orientation to these two definitions, we define the general term sustainable seafood consumption as “the consumption of sustainable amounts of seafood that was caught or farmed in ways that do not harm single species or ecosystems, so that today’s and future generations are equally able to benefit from marine resources”.

For consumers, the question still arises how this definition can be turned into actions. The sustainability of specific seafood products rises and falls with some criteria. In order not to harm single species or other parts of the marine ecosystem, variables like the stock status or the reproductive power need to be considered. At the same time, catching or farming methods, transportation, and packaging influence how sustainable a product is [16,17]. Most of these factors are constantly changing due to seasonal variations, climate change, technical improvements, governmental regulations, and market developments. At the moment of decision for or against a seafood product, consumers usually only have limited access to up-to-date sustainability criteria.

This challenge was addressed by the introduction of seafood labels [30,31] and seafood guides [32]. Seafood labels and seafood guides are designed to make sustainable seafood consumption more consumer-friendly by pre-classifying seafood according to sustainability criteria. We will discuss the feasibility, advantages, and disadvantages of these two approaches in the upcoming sections. Furthermore, alternative approaches like seafood consumption reduction will be discussed.

## 3. Seafood Labels

It is almost impossible for consumers to know which product fulfils all the criteria of seafood sustainability. This challenge is addressed by sustainability labels, making it possible to identify certified seafood at a glance. The most popular example in Europe is the Marine Stewardship Council (MSC) certification for wild caught fish. The blue MSC label marks products complying to two core principles: (1) sustainable fisheries standards and (2) chain of custody standards. The pendant to MSC for farmed fish is the Aquaculture Stewardship Council (ASC). Both MSC and ASC are charity organisations that certify fisheries according to independent expert assessments. In addition to MSC and ASC, there is a variety of labels for sustainable seafood like Friends of the Sea (FOS), Krav, or Global Trust Certifications, Ltd. (GTC).

The advantage for the consumer is that following labels is a relatively easy concept. Increased purchase of labelled products, in addition, encourages fishing and farming industries to achieve certification standards. In this case, sustainable utilisation of seafood is driven from bottom up. 

However, labelling also has weaknesses. Usually, supermarkets only provide a limited range of labelled products, which means reduced options to choose from for the consumer. Another weakness is that seafood labelling is not quick enough to follow the constantly changing stock conditions [33,34] and rarely includes CO_2_ emissions into its criteria [35]. Often, consumers are not able to explain the meaning of a label [36]. Furthermore, it was found that an aggregation of many different labels leads to consumer confusion and rejection [37]. Regarding raising awareness for marine resource conservation, the mere presence of labels does not influence conservation motivation or product choice [38]. 

As a consequence, simply introducing labels is not enough to make seafood consumption more sustainable; consumers also need to be informed about the existence and the meaning of labels and be motivated to pay attention to them [39,40]. 

## 4. Seafood Guides 

Institutions like the World Wildlife Fund (WWF), Greenpeace, the Marine Conservation Society, or Seafood Watch offer constantly updated, country-specific brochures or mobile phone apps to support sustainable seafood choices. Seafood guides provide some information on marine ecosystems, sustainable use of marine resources and consumer responsibility. Subsequently, a list of commonly used seafood from the particular country or region is presented. The seafood is listed according to a traffic light system coloured in green (recommended), yellow (be critical, think twice), and red (avoid). The categorization is made based on up-to-date scientific evidence, executed by the organisation itself or independent research institutions. 

It is an advantage that seafood guides offer a clear overview of the sustainability level of all major consumed seafood products of a country, together with some information. At the same time, it is a drawback that using seafood guides takes quite some time and effort. 

## 5. Alternatives to Seafood Labels and Seafood Guides 

Reducing seafood consumption and thereby taking off the pressure of marine ecosystems is another idea for responsible marine resource use [41]. However, it is, due to national and global economic dependencies, unrealistic that this could be realised in a conceivable time frame and without running economic and health-related consequences [42]. Another undesired side effect could be that the reduced consumption of one animal protein (e.g., seafood) might lead to the increased use of another animal protein (e.g., meat or dairy). As modern western diets include high percentages of animal protein, this is a likely consequence [43]. For the global goal of carbon emission reduction, replacing seafood with meat and dairy cannot be regarded as a solution. If seafood were to be replaced with plant based protein sources like seaweed or algae, the approach of reduction and replacement would lead to reduced fishing pressure and carbon emissions. However, this approach requires acceptance of new food sources as well as systemic changes in production and supply. Therefore, this option is interpreted as a prospective opportunity for future developments of the food market, but not as concrete behavioural advice we would suggest to seafood consumers today.

Another option for sustainable seafood consumption would be traditional fishing on a small scale. Private fishing is regarded as sustainable because it covers the immediate demand of a limited group of people, has low bycatch, and a very small negative effect on marine habitat. However, small-scale private fishing is not performed by a large number of consumers following the modern consumerism dominating in Western countries. Therefore, we focus on elaborating how to render processes embedded in typical western mass-consumption more sustainable. 

## 6. Barriers and Facilitators to Sustainable Seafood Consumption 

Knowing about barriers and facilitators of consumer behaviour supports the effectiveness of interventions towards sustainability [44]. However, most campaigns that are meant to encourage sustainable consumption exclusively focus on enhancing the consumer’s knowledge [45]. We state that concentrating on one factor is not enough. Purchase decisions are determined by a variety of psychological, contextual, and socioeconomic variables [46]. In regard to the complex nature of sustainable seafood consumption, we identified motivational variables that we believe to influence sustainable seafood consumption. Also, the socio-economic system in which consumers are embedded will be considered. 

## 7. Intentions

Intentions are essential for human behaviour because most behaviours are immediately preceded by an intention to act [47]. Intentions were found to precede sustainable consumption [48] and seafood consumption [49]. According to Ajzen [47], the stronger the behavioural intentions, the higher the willingness to try and the effort to overcome potential barriers. Intentions to sustainably consume seafood in the near future are therefore assumed to preceding sustainable seafood consumption directly. However, before forming the intention for an action, a corresponding motivation needs to be developed. This motivation highly depends on a person’s attitudes [50].

## 8. Attitudes

Attitudes are a “summary evaluation of a psychological object captured in such attribute dimensions like good-bad, harmful-beneficial, pleasant-unpleasant or likable-dislikeable” [51]. Positive and negative beliefs toward products (sustainably labelled seafood), consumption patterns (sustainable seafood consumption), or value systems (pro-environmentalism) form an individual’s attitudes. Attitudes around seafood consumption are mainly formed through beliefs about taste, distaste, nutritional value, ease of preparation, familiarity, and freshness [52]. Attitudes are further formed by experiences [53]. Positive experiences with sustainable seafood consumption are therefore assumed to create positive beliefs and to diminish negative beliefs. By this, scepticism toward new seafood products can be reduced [54].

People with strong pro-environmental attitudes have an increased probability to overcome barriers for pro-environmental actions in comparison to people with low general pro-environmental attitudes [55,56,57]. In turn, easy pro-environmental actions (switching off lights) are performed independent of people’s pro-environmental attitude, in comparison to difficult behaviours (installing solar panels), which are only performed by people who are very committed to environmental protection. We place sustainable seafood consumption in the medium-difficult range compared to other ecological behaviours and are therefore prone to be influenced by variation in attitudes [58,59,60]. 

## 9. Social Norms

Sherif [61] described social norms as “jointly negotiated rules for social behaviour”. Social norms are important for sustainable consumption [62] including seafood consumption [63] because consumers use the behaviour of other consumers as orientation for what they buy. It is relevant for sustainable seafood consumption that social norms vary to the extent to which they are injunctive or descriptive. The prescriptive power of these two sub categories can be contradictory: Injunctive norms are perceptions of what kind of behaviour is expected in certain situations, while descriptive norms represent the actual behaviour dominating this situation [64]. People might perceive sustainable seafood consumption as a desirable behaviour that is in line with marine conservation as a common norm (injunctive norm). At the same time, seafood is often perceived as a healthy and sustainable option compared to meat, which makes concern about seafood sustainability quite unusual (descriptive norm). 

The extent of how well sustainable seafood consumption is adopted by consumers thus might not only depend on individual motivation but also on the dominating behaviour of peers as well as perceived expectations. 

## 10. Knowledge

Many eco-campaigns are solely guided by the assumption that providing knowledge about environmental problems is the key to more environmentally friendly behaviour [65]. Until now, most approaches that focus on information only typically increase the level of knowledge but do not lead to the desired behaviour change [65,66,67,68]. For sustainable seafood consumption, two types of knowledge are relevant. Motivation to consume seafood responsibly is a consequence of knowledge about marine depletion. This is called background knowledge. Background knowledge was shown to increase the willingness to buy sustainable seafood [69,70]. However, knowing about problems is still not enough to make people act. They also need to know what kind of actions to perform. For sustainable seafood consumption, this procedural knowledge is particularly important. The actions that represent sustainable consumption of seafood are often unknown or unfamiliar, which potentially reduces action performance. Consumers who were familiar with the use of sustainable seafood labels were more willing to buy labelled products compared to consumers who were not familiar with them [71]. Both background and procedural knowledge are necessary but not sufficient preconditions for behaviour change toward sustainable seafood consumption.

## 11. Trust

Trust in the certification bodies is crucial, especially when choosing labelled seafood or seafood indicated as sustainable in a seafood guide are the behaviours representing sustainable seafood consumption. Consumers only accept paying more for a labelled product if they have trust in the third party giving the certification [72,73]. Throughout the last few years, many certification entities were criticized for fraud [74], especially in the seafood industry, which harmed consumer trust [33,75,76,77]. Only full transparency and strict compliance to sustainability standards can help certification systems to become and to stay credible [72,78,79,80]. 

## 12. Habits

The purchase of groceries is something highly habitual [81,82]. It is regularly repeated in similar temporal and regional conditions. This implies that buying food is a closed process: the same store, the same route to navigate through the shelves, the same range of products ending up in the basket [81,82]. The higher the level of automatization, the more difficult it becomes to change a behaviour [45,83,84]. Habits were found to predict sustainable consumption but also seafood consumption over and above some of the afore mentioned factors like attitudes and social norms [84,85,86,87,88,89,90]. We conclude that the shift from conventional to sustainable seafood consumption involves interrupting existing habits and creating new ones. To reach this goal, a high level of motivation and concretely planned actions are crucial, as are changes in the consumer environment.

## 13. Situational and Socioeconomic Conditions

Besides motivational factors, the situation an action is taking place in needs to be considered [91]. Situational conditions can be barriers as well as facilitators for pro-environmental actions [92] such as for sustainable consumption [93]. Given that motivational factors are present, the infrastructure still needs to provide opportunities for an action to be performed. If there are no sustainable seafood products available, product visibility is low, or product price too high, people can hardly reach their goal of consuming seafood responsibly. 

Availability was identified as the strongest situational factor predicting the consumption of organic food across countries [94,95,96]. Consumers cannot buy a product that is not offered to them. Consumers are also less likely to purchase sustainable seafood if it is perceived as hard to get [97,98,99]. Currently, Western supermarkets are dominated by a small range of products, and most of them come with their own concern in regards to sustainability. Therefore, availability of sustainable seafood products is crucial for the realisation of sustainable seafood consumption on a large scale. Good visibility and attractive appearance are, similar to availability, aspects that can facilitate or hinder the performance of sustainable seafood consumption [100]. People who do not intentionally search for sustainable seafood products can be steered to buy a sustainable product by its good visibility and attractive appearance. On the contrary, poor visibility and a rather unalluring look can impede the purchase of sustainable seafood, even in consumers who are motivated to buy them. High price premiums for sustainable—as compared to conventional—products were found to be a barrier for sustainable consumption and to lead to rejection [46,96,101,102,103]. At the same time, the influence of price is moderated by beliefs about quality and food safety [46]. In the case of seafood, the impact of price is ambivalent because here, choosing sustainable does not automatically imply paying more. Choosing sustainable seafood can mean choosing a different species (like Herring instead of Tuna) that often is cheaper. However, choosing sustainable seafood can also mean choosing a product carrying a label instead of the equivalent product without label. In these cases, the labelled product is usually more expensive. In these cases, consumers were found to tolerate a price premium up to 15% [71,104,105]. 

Besides availability, visibility, and price, time pressure is a factor that was found to impede the success of sustainable consumption. People pay less attention to product features like sustainability labels when they are short on time [106] and act more automatically [107]. Given that purchase situations often take place under time pressure (e.g., after work, on the way to somewhere), time pressure is suspected to be an important barrier.

National and international market developments, as well as political regulations, provide the overall frame for consumer behaviour and sustainable choice making [46,91,94]. The same accounts for sustainable seafood consumption. Availability and price of a certain seafood product are influenced by national and international trade but also changes in the ecosystem and which methods for fishing and farming are legalised and subsidised. 

Consumer choices depend on characteristics like gender, age, education, or the presence of children in the household [71,108,109,110]. The attempt to motivate sustainable seafood consumption behavior and socioeconomics are also relevant. It was shown that females dominate when it comes to both the purchase of organic products and of seafood [89]. Of course, this is potentially moderated by women often being in charge for grocery handling. People following a healthy or spiritual lifestyle and people with children purchase more organic products and more sustainable seafood [111,112,113]. Education, age, and income do not uniformly predict sustainable consumption [57,96,114,115,116]. Age and education were found to be related to the amount as well as the type of seafood consumed, with higher seafood consumption among older people and people with higher education [71,80,89]. The region of residence is a strong determinant of which type of seafood is consumed on a between- and within-country level [71,117]. 

To create a powerful intervention that motivates sustainable seafood consumption, we argue for taking into consideration the most relevant psychological factors, the socio-economic characteristics of the target group, and the situational frame the behaviour is going to take place in. We integrated all factors into one hypothetical model to illustrate how the single factors are assumed to interact with each other. 

## 14. Model of Sustainable Seafood Consumption

**Figure 1:**
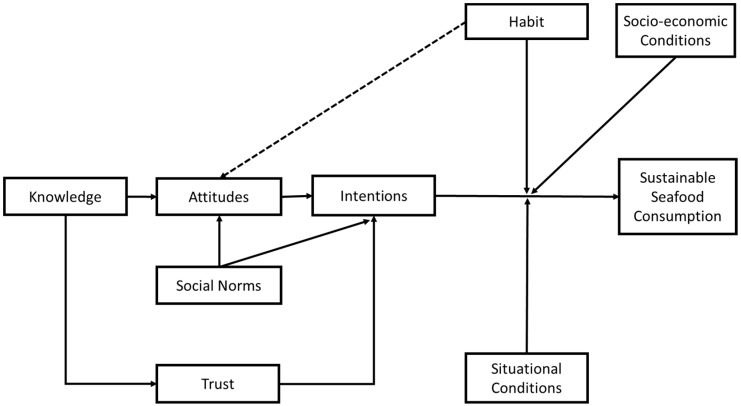
A comprehensive model for sustainable seafood consumption.

A hypothetical model was created, integrating all afore mentioned variables (intentions, attitudes, social norms, trust, knowledge, habits, situational and socioeconomic conditions, see Figure 1). The variables were arranged according to their role in regards to our target behaviour, sustainable seafood consumption. In orientation to well-established models like the Theory of Planned Behaviour (TPB, [47]), the Motivation-Opportunity-Ability Model (MOA [93]), and the Comprehensive Action Determination Model (CADM, [87]) the variable structure was adapted. 

The dependent variable reflects our target behaviour, sustainable seafood consumption, which can be performed in different ways (using seafood labels, seafood guides, etc.). Directly preceding this behaviour are intentions. Intentions typically precede behaviours that are performed consciously and that are the result of a motivation formed earlier [47]. This relationship between intentions and behaviour is moderated by habits [87,88,118]: the intention-behaviour link is stronger the more deliberate, and the less automatized a behaviour is. In the case of an automatism, less cognitive effort is required, and the behaviour is triggered by situational cues [82]. We suggest that the performance of sustainable seafood consumption can be the result of both the intention to consume seafood more sustainable or a habit around (sea-) food consumption. Existing, unsustainable habits around seafood are particularly difficult to overcome because habits lead to ignorance of new information [119] and overestimation of disadvantages of alternative behaviours [120]. The link between intentions and behaviour is moderated by two more variables: the situational and socioeconomic conditions that build the frame for actions [87,88,93,118]. In line with the MOA, situational conditions can facilitate or hinder behaviour [93] by providing good or bad opportunities to act. The socioeconomic conditions reflect, together with habits, important characteristics of a person and that person’s ability. Habits moderate the relationship between intentions and behaviour and also influence attitudes throughout the process of behaviour change (illustrated by the dashed arrow). First, contradictory habits are a barrier to the establishment of a new behaviour, but as soon as a new behaviour is repeated more regularly, it becomes habitual, demands less cognitive effort, and likely becomes the preferred behavioral alternative because reduced cognitive effort leads to more positive attitudes [121]. Attitudes influence behaviour mediated by intentions [47]. The more positive attitudes are associated with sustainable seafood consumption, the more probable it becomes that behavioural intentions are formed [56,60]. Like attitudes, social norms are supposed to influence behaviour indirectly across intentions [47]. The perception of sustainable seafood consumption being popular among peers is assumed to increase the probability that intentions for this behaviour are formed. Knowledge predicts attitudes and trust. Located further back in the model, it resembles a necessary but not sufficient predictor. Knowledge about the importance of sustainable seafood consumption creates positive attitudes toward this behaviour. The same accounts for procedural knowledge about the actions that lead to a responsible consumption of seafood. Knowledge of the existence and the meaning of labels is supposed to influence the level of trust. Trust in the third parties that support consumers in sustainable seafood consumption by providing labels or guides is associated with intentions. Even if consumers have positive attitudes on consuming seafood sustainably, a lack of trust in the certification bodies is a barrier for the formation of concrete intentions (to use seafood labels for example).

Theoretically modelling sustainable seafood consumption and its predictors helps to understand how consumers’ decisions are made. It outlines which factors precede others and how they interact with each other. From an interventional perspective, potential levers and directions can be identified by using models. This saves valuable resources like time and money and increases efficiency. Our next step is the identification of the most important predictors within our model and cross validating them on different samples. This gives an idea of generalizability and potential differences between target groups. Environmental psychology research provides literature on interventions based on predictive variables [122,123], which will be used as inspiration for intervention design. 

Theoretically proposing complex models comes with certain limits, mainly due to the exploratory stage in which theoretical models are located within the research process. Theoretically constructing a model is only the beginning of model selection and ideally serves as an approximation to the data generation process [124]. Experimental studies then help confirm the relationships between single variables and the model structure as a whole. Models like this, which integrate many variables, might suffer from poor fitting propensity [125], and often, more parsimonious structures might provide a better fit [126]. It is likely that after careful cross-validation on real data, some variables need to be relocated within the model or removed. Our overall goal with this research in the long-term is not forcibly fitting all the proposed variables into one model, but identifying the variables that strongly predict sustainable seafood consumption in order to design powerful interventions.

## 15. Conclusions

Our main conclusion after this literature review is that sustainable seafood consumption is a challenge for the consumer. The concept itself is fuzzy, and the application of it requires cognitive effort and the interruption of current habits. Using seafood carrying the MSC label as an approximation of sustainable seafood consumption levels, only 12% of all seafood meets the MSC criteria [127]. This leaves a lot of room for improvement, both in Europe and worldwide. Another critical issue is that the focus lies on a small number of mainly unsustainable species that are commonly consumed as a part of Western meals, all of them coming with their own concern with regard to sustainability. Consumers do not always make purchase decisions consciously: instead, they are driven by automated procedures or follow the behaviour of their peers. Past research on pro-environmental behaviour showed further that depending on the specific behaviour stimulated, the target group addressed, and the environmental conditions, the impact of motivational variables varies in strength and influence [66,123,128], which will be an interesting area to look at in the area of seafood consumption. The ultimate performance of sustainable seafood consumption is the consequence of an interaction of motivational, situational, and socioeconomic factors. 

The model of sustainable seafood consumption adds value to the present research on sustainable marine resource use. The model delivers an individual’s perspective in addition to the socioeconomic framework. The focus on motivational variables offers a stimulating approach for the realisation of changes in the seafood consumption sector. Psychology becomes increasingly important in strategy development for sustainability [129]. This model provides potential levers to be pulled to motivate consumers for sustainable purchase decisions and thereby influence the seafood industry from the bottom up.
